# Visualization of GaN surface potential using terahertz emission enhanced by local defects

**DOI:** 10.1038/srep13860

**Published:** 2015-09-09

**Authors:** Yuji Sakai, Iwao Kawayama, Hidetoshi Nakanishi, Masayoshi Tonouchi

**Affiliations:** 1 Institute of Laser Engineering, Osaka University, Suita, Osaka 565-0871, Japan; 2 SCREEN Holdings Co., Ltd., Kyoto 612-8486, Japan

## Abstract

Wide-gap semiconductors have received significant attention for their advantages over existing semiconductors in energy-efficient power devices. To realize stable and reliable wide-gap semiconductor devices, the basic physical properties, such as the electric properties on the surface and at the interface, should be revealed. Here, we report visualization of terahertz (THz) emission from the surface of GaN, which is excited by ultraviolet femtosecond laser pulses. We found that the THz emission is enhanced by defects related to yellow luminescence, and this phenomenon is explained through the modification of band structures in the surface depletion layer owing to trapped electrons at defect sites. Our results demonstrate that the surface potential in a GaN surface could be detected by laser-induced THz emission. Moreover, this method enables feasible evaluation of the distribution of non-radiative defects, which are undetectable with photoluminescence, and it contributes to the realization normally-off GaN devices.

As a material for next generation devices, such as light sources, high-power devices, and solar cells, wide-gap semiconductors have attracted significant attention over the years. Blue light sources manufactured from gallium nitride (GaN), which is a typical wide-gap semiconductor, have already been used in many industrial applications. For higher luminous efficiency, the crystal quality of GaN must be improved. In addition, power devices made from GaN have many superiorities over existing power devices, e.g., high-frequency operation, break-down field, and heat conductivity, however there are still some problems to be solved such as a current collapse^1^ and a normally-on property^2^. Among these, the normally-on characteristics, i.e., a high electric field exists without external bias voltage due to polarization^3^ in the interface of the hetero-junction, have been a serious problem for practical applications. Therefore, control of the surface and the interface polarization is required. However, there is no convenient method for measuring the “local” polarization without electrical contacts.

On the other hand, terahertz (THz) spectroscopy is expected to evaluate semiconductor materials and devices. The optical and electrical properties of various semiconductors have been investigated by THz time-domain spectroscopy^4^,^5^,^6^ and by laser-induced THz emission spectroscopy^7^,^8^,^9^. The laser THz emission microscope (LTEM), which images the amplitude of THz emission from materials and devices, is also used for evaluating semiconductor devices^10^,^11^.

Here, we measured THz emission from GaN surfaces excited by ultraviolet (UV) femtosecond laser pulses and found enhancement of THz emission due to defects in the GaN surface by comparing the photoluminescence (PL) measurements. The enhancement is explained by modification of the band bending induced by the accumulated negative charges in the surface depletion layer. Accordingly, the present results suggest that LTEM is capable of measuring not only the defect density but also the local surface potential.

## Results

**Figure 1:**
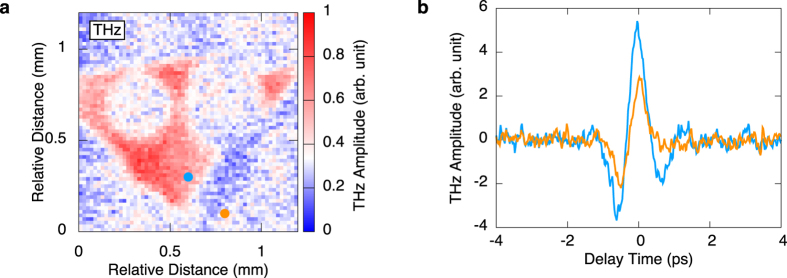
(**a**) The LTEM image of n-type GaN with an excitation wavelength of 260 nm. (**b**) The THz waveforms at two points in (**a**). Line colors correspond to point colors in (**a**).

Figure 1a,b respectively show the LTEM image of the Ga-polar (0001) surface of n-type GaN, which was provided by Sumitomo Electric Industries, and the waveforms of THz emissions at the two points, which are shown as sky blue and orange marks in the LTEM image. All the measurements in this study were performed at room temperature. The excitation wavelength and the photon density per pulse are 260 nm and 3.4 × 10^13^/cm^2^, respectively. Because the band gap energy of GaN is 3.4 eV, the incident photon energy (4.8 eV) is sufficient to excite an electron from the valence band to the conduction band. The LTEM image corresponds to the mapping of the negative peak amplitude at 0 ps, as shown in Fig. 1b.

As shown in Fig. 1a, a noticeable distribution of the THz amplitude in the plane can be observed in the LTEM image, even though pulse widths were almost unchanged.

**Figure 2:**
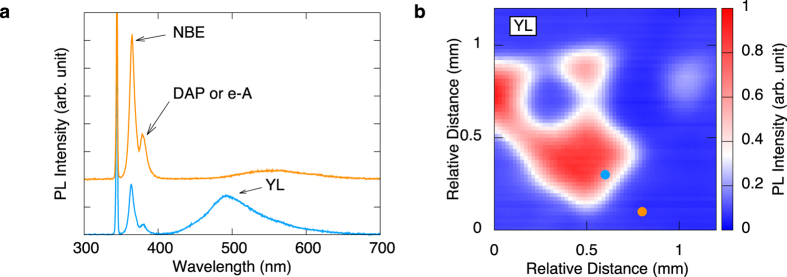
(**a**) The PL spectra of GaN at two characteristic points shown in Fig. 2 (**b**) as blue and orange points. The excitation wavelength is 345 nm. (**b**) PL 2D mapping using the intensity of YL peak with the excitation wavelength of 345 nm.

Figure 2a shows PL spectra measured at two points in the same GaN sample. The PL measurement was performed at room temperature, and the excitation wavelength and photon density per pulse were 345 nm and 4.8 × 10^13^/cm^2^, respectively. These spectra contain the excitation source at 345 nm, the near-band-edge (NBE) emission peak at 365 nm, the luminescence peak by donor-acceptor pair (DAP) or conduction-band-to-acceptor (e -A) transition at 390 nm, and the broad luminescence around 500 nm, corresponding to photon energy of 2.2–2.4 eV. This broad luminescence is called “yellow luminescence” (YL) and is primarily attributed to defects that function as deep acceptors. However, the origin of the deep acceptor has not been determined, and some models have been proposed, e.g., Ga vacancy, *V*_Ga_^12^,^13^,^14^, carbon substituting nitrogen, C_N_^15^, and C_N_-O_N_ complex^16^. The intensities of YL depend on measured points on the GaN surface, which are depicted by a sky blue line and an orange line in Fig. 2a, seem to reflect the density of defects on the GaN surface. To clarify the distribution of YL intensity, we have performed PL intensity mapping with YL of the GaN in the same area as the THz mapping in Fig. 1a. The YL intensity mapping shown in Fig. 2b has almost the same contrast as the LTEM image. Thus, the intensity of THz emission from the region with high defect density (HDD) is higher than that from the region with low defect density (LDD). The results suggest that an enhancement of the THz emission is induced by the defects in the GaN surface.

**Figure 3:**
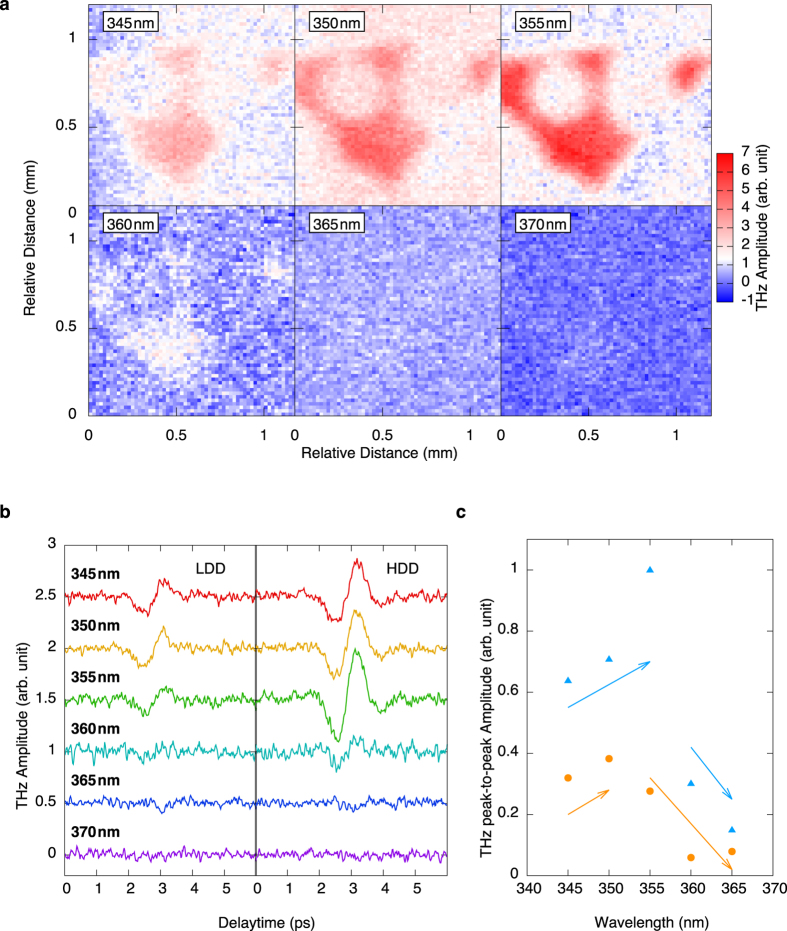
(**a**) The LTEM images generated by various excitation wavelengths around the band gap energy of GaN. The band gap energy corresponds approximately to 365 nm. Note that offsets were added to waveforms below 370 nm. (**b**) The THz waveforms at the LDD region and the HDD region for each excitation wavelength. (**c**) Peak-to-peak amplitude of the THz emission at each excitation wavelength. Circle and triangle represent the peak-to-peak amplitude in the LDD and the HDD region, respectively. All values are normalized by the peak-to-peak amplitude at 355 nm in the HDD region. Arrows are provided to guide the eyes.

Figure 3a shows LTEM images excited by the UV laser with various wavelengths around 365 nm, corresponding to the band gap energy of GaN under constant photon density conditions (4.9 × 10^13^/cm^2^). We also measured waveforms of THz radiation from HDD and LDD regions in the n-type GaN surface with various excitation wavelengths, as shown in Fig. 3b. The THz peak amplitudes at 3 ps (the negative peaks) in Fig. 3b were used to obtain the LTEM images. When the excitation photon energy is above the band gap energy (that is, the excitation wavelength is below 365 nm), we can clearly distinguish the HDD region from the LDD region, as shown in Fig. 3a. However, the contrast of the image rapidly decreases under the excitation by the 360 nm wavelength, and the HDD region is indistinguishable from the LDD region when the wavelengths of the excitation laser are 365 and 370 nm, namely, when the photon energies are below the band gap energy.

Figure 3c shows the wavelength dependence of the peak-to-peak amplitudes of the THz emissions from the LDD and HDD regions shown in Fig. 3b. The peak-to-peak amplitudes of the THz emissions from both the HDD and LDD region increase with increasing excitation wavelength up to 350 nm. Though the THz peak-to-peak amplitude in the HDD region still increases, that in the LDD region begins to decrease when the wavelength is 355 nm. All THz signals rapidly decrease when the wavelengths are above 360 nm.

## Discussion

**Figure 4:**
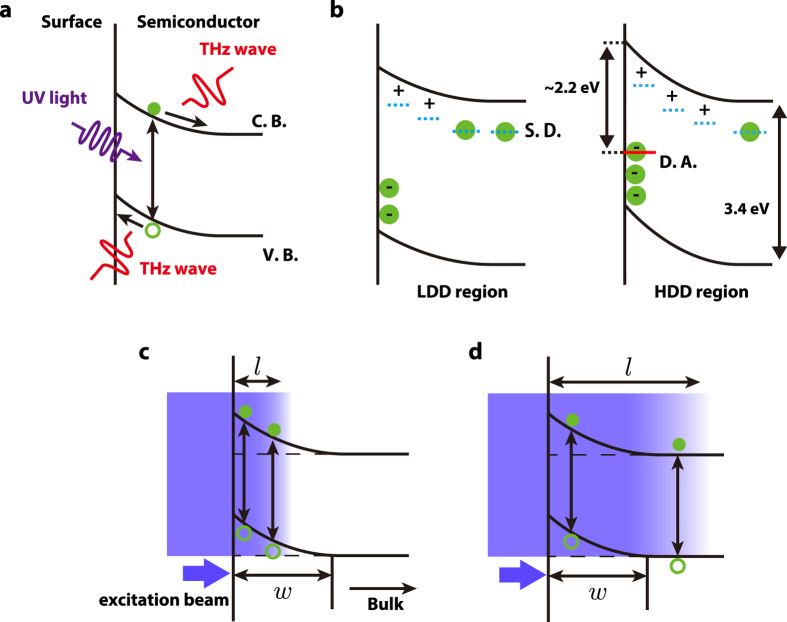
(**a**) Schematic illustration of the general THz emission process in wide-gap semiconductors, where C. B. and V. B. represent the conduction band and valence band, respectively. Electrons and holes are described with open and filled circles, respectively. (**b**) Schematic illustration of the band structure near the surface in the LDD region (the left panel) and in the HDD region (the right panel). Blue dash lines and red line represent shallow donor levels (S.D.) and deep acceptor level (D.A.) caused by YL-related defects, respectively. (**c**,**d**) Schematic illustration of the relation between the thickness of the depletion layer, *w*, and the penetration length, *l*.

Several mechanisms for exciting THz wave generation from semiconductor surfaces using femtosecond laser pulses have been proposed, e.g., the current surge effect^17^, photo-Dember effect^18^, and optical rectification^19^. The result that THz signals rapidly decrease under excitation with wavelengths above 360 nm, shown in Fig. 3, indicates that THz radiation is triggered by photoexcited carriers that are excited from the valence band to the conduction band. Therefore, optical rectification is not a valid radiation mechanism in our experiment. The photo-Dember effect is dominant for a narrow band gap semiconductor such as InAs; however, THz emission from the surfaces of semiconductors with wider band gaps such as GaAs and InP originates from the current surge effect^20^. The band bending for n-type and p-type GaN is upward and downward, respectively. Wu *et al.* reported that the surface barrier of n-type GaN grown on SiC was 0.75 ± 0.1 eV with upward band bending^21^. Therefore, the mechanism of THz radiation from n-type GaN is primarily considered to be the current surge effect, as shown in Fig. 4a.

Left and right panels in Fig. 4b show schematic illustrations of band structures near the surface in the equilibrium state of the LDD and HDD regions, respectively. As shown in the left panel, electrons are generally trapped at surface states or shallow donor levels (S.D.), which are shown as blue dash lines in Fig. 4b. The YL-related defects act as deep acceptors whose level (D.A.) is shown as a red line in the right panel. Because this level is approximately 2.2 eV below the minimum of the conduction band^14^, deep acceptor levels in n-type GaN are almost fully occupied by electrons in the equilibrium state as shown in the right panel. Therefore, the negative charge density at the surface increases with increasing the number of deep acceptors. By charge conservation, the positive charge density in the depletion layer also increases. Since the surface potential is increased by the negative space charge, the surface potential in the HDD region is larger than that in the LDD region as shown in Fig. 4b. The correlation between the surface barrier and YL intensity in GaN, which was measured by Kelvin force techniques, has also been reported by Shalish *et al.*^22^. Consequently, the increase of surface potential causes the enhancement of THz emission.

Additionally, the wavelength dependence of the intensity of THz radiation, which is shown in Fig. 3c, can be explained by competition between the penetration length of an excitation laser, *l*, and the thickness of the depletion layer in the GaN surface, *w*, as shown in Fig. 4c,d. There is a significant change in the absorption coefficient around the band gap energy^23^,^24^, and hence the penetration length, *l*, rapidly increases with increasing wavelength (*l* ≃ 30 nm for 3.5 eV and *l* ≃ 100 nm for 3.45 eV in ref. 23). The thickness of the depletion layer in a n-type semiconductor, *w*, is represented by the following equation:


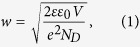


where *ε* is the static dielectric constant, *ε*_0_ is the vacuum permittivity, *V* is the surface barrier height, *e* is the electron charge, and *N*_*D*_ is the concentration of donors. For the n-type GaN with *N*_*D*_ ~ 10^17^–10^18^ cm^−3^ used in this experiment, *w* becomes 30–100 nm^25^. When *l* < *w* is satisfied (Fig. 4c) in the shorter wavelength range, the photocurrent carriers increase with increasing wavelength, which means that *l* increases, because the photoexcited carriers in the layer very close to the surface decay immediately due to much shorter surface recombination lifetime than that in bulk. Therefore, the THz amplitudes increase with increasing the excitation wavelength. When *l* > *w*, i.e., the longer wavelength range as shown in Fig. 4d, the photocurrent decreases with increasing wavelength because the photoexcited carriers generated outside the depletion layer increase and have little contribution to the photocurrent due to weak electric field to accelerate carriers. On the other hand, *w*, which depends on *N*_*D*_ as shown equation (1), in the HDD region should be thicker than that in the LDD region because the density of positive charge in the depletion layer is estimated as *N*_*D*_ − *N*_*A*_, where *N*_*A*_ is the density of acceptors. Therefore, the THz emission intensity starts to decrease at the wavelength of 355 nm in the LDD region, while the intensity of THz radiation continues increasing in the HDD region. Consequently, the LTEM image at 355 nm has the highest contrast.

As explained above, we confidently conclude that LTEM is able to evaluate the relative surface potential. However, further studies are required in order to discuss the sensitivity and the quantitative value of teh surface potential.

We further consider the possibility that the additional photoexcited carriers excited from the defect levels near the direct interband excitation enhance the THz radiation. This additional excitation from the defect levels should be induced by photons with a lower energy than the band gap energy in the HDD region. However, the THz signal rapidly decreases and becomes almost zero when the photon energy of the excitation laser pulse is below the band gap energy, as shown in Fig. 3. Under high power excitation where the photon density per pulse is 1.9 –2.0 × 10^14^/cm^2^ (more than quintuple the power of the present measurement), no emission is observed in the LDD region at 370 nm or less than 375 nm (see the supplementary information). Therefore, the carrier excitation from the YL-related defect levels has little contribution to the THz radiation.

In summary, we have measured the THz emission from GaN excited by UV pulsed laser and performed two-dimensional (2D) mappings with the intensity of the THz emission. Comparing the LTEM image with PL 2D image, the THz emission was clearly enhanced by YL-related defects in the GaN surface. The result of THz emissions generated by various excitation wavelengths suggests that the enhancement of THz emission can be explained by the change of surface potential induced by deep acceptors consisting of YL-related defects. Our results demonstrate that LTEM can evaluate not only the relative density of the defect as well as PL but also the “local” surface potential of wide-gap semiconductors. This method can evaluate non-radiative defects, which are undetectable with PL and will be valuable for realizing normally-off GaN devices.

## Methods

The second and third harmonics of a Ti:sapphire laser were used as the LTEM excitation source with a center wavelength of 690–780 nm, a pulse width of about 100 fs and a repetition rate of 80 MHz. The crystals used for the second harmonic generation and third harmonic generation were barium borate (BBO) and bismuth borate (BIBO) crystals, respectively. The second or third harmonic was focused on the sample by a 20X objective lens. The beam power, which was incident on the objective lens, was several tens of milliwatts, and the spot diameter on the sample was about 50 μm. The generated terahertz wave was focused on the detector by a pair of parabolic mirrors and a silicon semispherical lens. A dipole-type photoconductive antenna (PCA) with a LT-GaAs substrate was used for the detector. The fundamental beam of the Ti:sapphire laser was used to generate photocarrier on the PCA, and its power was about 6 mW. The PL was detected with a fiber optic spectrometer, USB 2000 + (Ocean Optics).

## Additional Information

**How to cite this article**: Sakai, Y. *et al.* Visualization of GaN surface potential using terahertz emission enhanced by local defects. *Sci. Rep.* **5**, 13860; doi: 10.1038/srep13860 (2015).
